# KNOWLEDGE OF PUERPERAL MOTHERS ABOUT THE GUTHRIE TEST

**DOI:** 10.1590/1984-0462/;2017;35;2;00010

**Published:** 2017-05-15

**Authors:** Giovanna Abadia Oliveira Arduini, Marly Aparecida Spadotto Balarin, Roseane Lopes da Silva-Grecco, Alessandra Bernadete Trovó de Marqui

**Affiliations:** a Universidade Federal do Triângulo Mineiro (UFTM), Uberaba, MG, Brasil.

**Keywords:** Pediatrics, neonatal screening, health education

## Abstract

**Objective:**

This study aimed to assess the knowledge of puerperal mothers about the Guthrie test.

**Methods:**

A total of 75 mothers who sought primary care between October 2014 and February 2015 were investigated. The form was applied by the main researcher and the data was analyzed, using descriptive statistics with Microsoft Office Excel, and *Statistical Package for Social Sciences* (SPSS) programs. Association tests and statistical power were applied.

**Results:**

Among the 75 mothers, 47 (62.7%) would have liked to receive more information about the newborn screening, especially regarding the correct sample collection period, followed by the screened morbidities. Most participants (n=55; 85.9%) took their children to be tested between the third and the seventh day of birth, as recommended by the Brazilian Health Ministry. Fifty-four women (72%) were unable to name the morbidities screened by the test in Minas Gerais, and they were also unaware that most have genetic etiology. The health professional who informed the mother about the Guthrie test was mainly the physician. This information was given prenatally to 57% of the cases, and to 43 % at the time of discharge from the hospital. The association test showed that mothers with higher education have more knowledge about the purpose and importance of the Guthrie test. The statistical power was 83.5%.

**Conclusions:**

Maternal knowledge about the Guthrie test is superficial and may reflect the health team’s usual practice.

## RESUMO

**Objetivo:**

Identificar o conhecimento das puérperas sobre o teste do pezinho.

**Métodos:**

Foram investigadas 75 puérperas que buscaram atendimento na atenção primária no período de outubro de 2014 a fevereiro de 2015. Um formulário foi aplicado pela pesquisadora principal e os dados resultantes foram analisados por estatística descritiva por meio dos programas Microsoft Office Excel e *Statistical Package for Social Sciences* (SPSS). Foi feito teste de associação e determinado o poder estatístico.

**Resultados:**

Das 75 puérperas, 47 (62,7%) gostariam de receber maiores esclarecimentos sobre o teste do pezinho, com destaque para o período adequado de coleta, seguido pelas doenças triadas. A maioria das participantes (n=55; 85,9%) levou seu filho para realização do teste entre o terceiro e o sétimo dia de vida, conforme preconizado pelo Ministério da Saúde. Das puérperas, 54 (72%) não souberam citar quais são as doenças triadas pelo teste do pezinho em Minas Gerais nem sabiam que a maioria delas tem etiologia genética. O profissional de saúde que informou sobre esse teste foi um médico. Essa orientação ocorreu durante o pré-natal em 57% dos casos e, na alta hospitalar em 43%. O teste de associação mostrou que as mães com maior escolaridade possuem maior conhecimento sobre a finalidade e a importância do teste do pezinho. O poder estatístico encontrado foi de 83,5%.

**Conclusões:**

O conhecimento das puérperas sobre o teste do pezinho é superficial e pode ser reflexo da atuação da equipe de saúde.

## INTRODUCTION

The Guthrie test, included in the Neonatal Screening Program (NNSP), aims at detecting infectious diseases and genetic characteristics, especially inborn errors of metabolism, asymptomatic at birth. This examination enables the early diagnosis and treatment of the patients with these diseases, in order to avoid damage to the child, such as intellectual disabilities.^1^ ^,^ ^2^ ^,^ ^3^ For the Guthrie test a few drops of blood are taken from the heel of the newborn (NB), and collected on filter paper. The correct period for the sample collection should not be less than 48 hours of protein feeding and should not exceed 30 days from birth; however, the ideal period would be between the third and seventh day of birth in newborns.^2^

In Brazil, the NNSP was regulated by Decree No. 822 of June 6, 2001, and deployed in three phases, according to the level of organization and coverage of each state, as a possibility to trace four diseases.^4^ In 2012, the fourth phase was deployed, which included the additional screening for two more disorders, thus totaling six.^5^ The State of Minas Gerais, Southeastern Brazil, was found in phase IV of the NNSP and identifies six diseases: phenylketonuria (PKU), congenital hypothyroidism (CH), hemoglobinopathies (Hb), cystic fibrosis (CF), congenital adrenal hyperplasia (CAH) and biotinidase deficiency (BD).^4^ ^,^ ^5^ The good prognosis of the entities identified by the Guthrie test depends on early diagnosis, treatment, and monitoring since the first months of the birth.^6^ It is worth noting that in order to include the diseases to be investigated in neonatal screening (NS), the following requirements must be met: if untreated, the disorder causes serious consequences for the health of the affected; there is a treatment that can substantially modify the natural history of the disease; the treatment is significantly more effective when deployed in preclinical phase of disease; there should be a screening test which is simple, efficient, and applicable on a large scale and low cost, and; the disorder should be common enough.^3^

The diseases detected by the Guthrie test show varied incidences, mostly in some Brazilian states. In the city of Maringá, Paraná state, in the period from 2001 to 2006, the incidence in relation to the number of live births (LB), were PKU - 1:20,529; CH - 1:2,281; Hb - 1:3,421; CF - 1:10,264 and BD - 1:6,843 of LB. In Santa Catarina, between the years 2004 and 2006, the following incidences were reported: PKU - 1:28,862, CH - 1:2,876, CF - 1:5,121, Hb - 1:14,446, and for CAH - 1:11,655 of LB.^8^ The incidence of PKU was 1:28,309 and 1:33,068 of LB for the states of Tocantins^9^ and Mato Grosso,^10^ respectively. In those states, incidence of 1:4,632^9^ and 1:9,448^10^ LB were reported to CH. The Neonatal Screening Program of the Clinical Hospital of the School of Medicine of Ribeirão Preto (HCRP) at the *Universidade de São Paulo* (USP), Brazil, declared incidence of 1:2,595, 1:19,409 and 1:4,120 LB for CH, PKU, and Hb, respectively.^11^

The Guthrie test represents an action of preventive pediatrics, and the largest initiative of the Unified Health System (SUS) in the area of genetics. It is characterized by five steps: universal screening, active search, conducting diagnostic tests, treatment, and periodic evaluation of the system. In the first step, all infants should be screened and the actions of the nursing team, the obstetrician, and pediatrician are fundamental. These professionals are responsible for the guidance to parents regarding the existence of the Guthrie test, the benefits of early detection of diseases to be sorted and what they are, the risks for the newborn not submitted to the test, the appropriate age for its implementation, the need for diagnostic tests for those who are positive in the screening, the possibility of false positive and false negative and the process of monitoring and receiving the results. The second stage comprises the active search, characterized by monitoring results and by the location of the newborn and his family, especially if the result of the screening is changed. In the third step diagnostic tests are performed in which positive results are differentiated from the negative results. The treatment is the fourth step, usually carried out throughout life, and monitored by a multidisciplinary team of referral service in Neonatal Screening. The fifth step is a critical evaluation of the system, which should be constant because it allows analyzing the effectiveness of NS.^3^ Some published studies have shown low understanding of the theme Guthrie test, both among health professionals^12^ ^,^ ^13^ and parents.^6^ ^,^ ^14^ ^,^ ^15^ ^,^ ^16^ ^,^ ^17^ ^,^ ^18^

This study presents the following guiding question: what do mothers know about Guthrie test? Considering that mothers are in charge of taking care of newborns, this study is justified by the importance of measuring their knowledge about the Guthrie test, because only a correct understanding, and in a timely manner, will encourage them to take their children to the sample collection event in the appropriate period, which would avoid subsequent sequels, especially the intellectual disabilities. Added to this, the inflow of appropriate information will influence positively mother’s behavior to ensure the promotion of health and the welfare of their children. Thus, the aim of this study was to identify the knowledge of the puerperal women about the Guthrie test.

## METHOD

This is a study of the descriptive, cross-sectional, and quantitative approach. The data were obtained after approval of the project by the Human Research Ethics Committee at the *Universidade Federal do Triângulo Mineiro* (UFTM), under opinion number 853,544. This study, which involved humans, followed the criteria of Resolution no. 466/2012 of the National Health Council. The data was collected form October 2014 to February 2015, at the Center for Comprehensive Women’s Healthcare of the municipality of the state of Minas Gerais.

The inclusion criteria were as follows: a woman over 18 years of age who had given birth recently and her child was up to 40 days of age. In addition, she had accepted to participate in the research by signing the informed consent form (ICF). The age of the neonate was based on the fact that the postpartum period lasts approximately six weeks after the birth and, in this short interval, the information provided by the mothers would be more reliable due to the proximity of the period recommended by the Ministry of Health (MOH) to perform the examination. Therefore, we included 75 women who fulfilled the inclusion criteria and who sought medical care in primary care, especially for aid in the management of breastfeeding during the data collection period. Those who were excluded did not meet these criteria.

A form for data collection was elaborated by the researchers, using as a model based on the results of scientific articles that have been published on this topic and validated among members of our research group. The choice of the form is justified because the questionnaire was filled out by the researcher and had greater acceptance, as the mother had her son in her arms. The same person administered the questionnaire to all women who had recently delivered. The questionnaire contained closed questions, some dichotomous variables (“yes” or “no”) and other of multiple-choice. The open questions were restricted to personal information of the mother - age and contact telephone number - and of the neonate - dates of birth of the child and data collection. The form was composed of two parts: sociodemographic characterization and understanding of the puerperal women about the Guthrie test. The interviewees had its identity preserved, being identified by means of numbers.

The data were tabulated and analyzed by means of descriptive statistics with the programs Microsoft Office Excel and the Statistical Package for Social Sciences (SPSS) version 21.0. To analyze the association between age, education, number of children, and income with the correct answers for purpose and importance of the Guthrie test, the chi-square test was used, adopting the significance level of α=0.05. In order to determine the statistical power, with the sample size of 75 mothers, the variables importance of the test and education were used, and the significance level of α=0.05 was applied.

## RESULTS

Table 1 shows the sociodemographic characterization of the puerperal women investigated. With respect to the knowledge of the interviewees on the Guthrie test, the results obtained for this category are shown in Tables 2 and 3. Almost all (74; 98.7%) women are aware of the importance of the test for their child, and the majority (64; 85.3%) already performed the examination on their sons, whereas few (11; 14.7%) are yet to do so. Although most of them believe the test is important, 15 participants reported not knowing the importance, or that the Guthrie test is a routine examination. In relation to the health professional who provided information about the Guthrie test, the majority mentioned a doctor - cited by 45 women (60%) - followed by nursing staff (nursing assistant, licensed practical nurses, and nurses) - mentioned by 30 of them (40%). When questioned about the diseases screened through the Guthrie test, 54 (72%) did not know how to quote them. Only few women partially reported the diseases that are detected by this test. These results are presented in Table 4. With regard to the occasion the puerperal women received guidelines, 57% reported having received them during the prenatal care and 43% at hospital discharge. The quality of the information received was considered good for 45% of them.

**Table 1:**
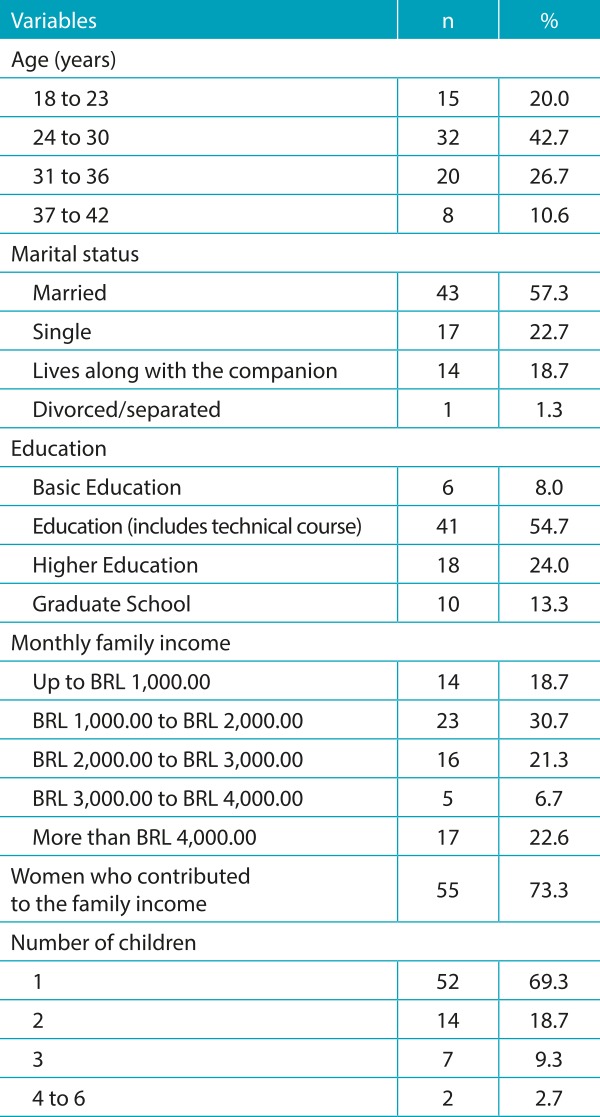
Sociodemographic characterization of 75 puerperal women investigated.

**Table 2:**
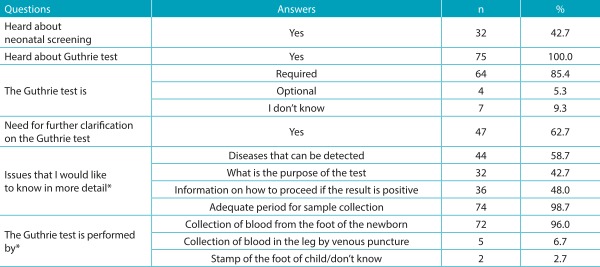
Women’s knowledge of the Guthrie test.

%: in relation to the total number of responses in each variable: Guthrie test; NS: neonatal screening; *in this question, the mother could indicate more than one answer.

**Table 3:**
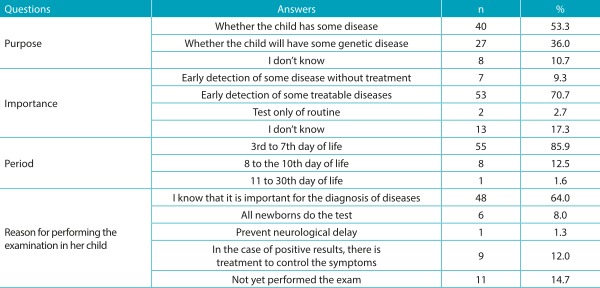
Women’s knowledge of the purpose, the importance, the period of completion, and reason that led her to perform the Guthrie test in his son.

%: in relation to the total number of responses in each variable.

**Table 4:**
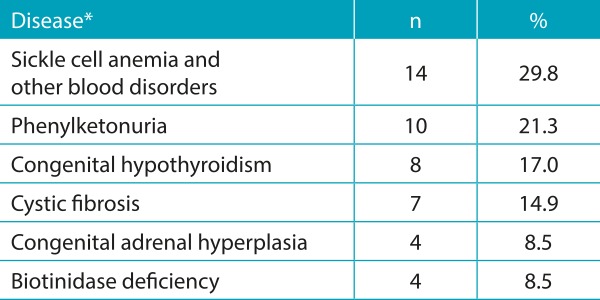
Diseases investigated by Guthrie test, according to the report of the puerperal women.

*in this matter, the mother could indicate more than one answer, so the total number is 21, which would correspond to those women who knew what are the diseases detected by the Guthrie test.

In the analysis of the association of the knowledge of mothers about the purpose and importance of the Guthrie test, with the variables such as age, number of children, income, and schooling, only the latter exhibited statistical significance. With respect to the purpose of the Guthrie test, it was observed that, of the 47 women with secondary education, 11 (23.4%) of them answered correctly to the questions, whereas among 28 women who were more educated, 16 (57.1%) answered correctly, thereby indicating a significant association (*p*=0.003). With regards to the importance of the Guthrie test, among 47 interviewees who had completed secondary education, 28 (59.6%) of them answered correctly and, among the 28 with higher education or more, 25 (89.3%) answered correctly, thereby indicating again a significant association (*p*=0.006). In other words, more educated mothers have greater knowledge about the purpose and importance of the Guthrie test. The statistical power was found to be 83.5%, which is appropriate to the standards commonly used for determining the sample size.

## DISCUSSION

The scientific literature generally refers to neonatal screening as synonymous to the Guthrie test, ^15^ ^,^ ^17^ ^,^ ^18^ ^,^ ^19^ and this reveals a misunderstanding in the definition of those terms. The Guthrie test is one of the tests included in the NNSP, which also covers the hearing screening test, the vision screening or the red reflex examination, cardiac screening or pulse oximetry, and the lingual frenulum evaluation. All mothers have heard about the Guthrie test, thus corroborating the data available in the literature.^14^ ^,^ ^17^ ^,^ ^19^ However, most people are not aware of NS, which show thereby the need to clarify to these mothers the association between both.

With regards to the test characteristics, the majority of the interviewees were aware it is mandatory. This obligation was established by Law no. 8.069/1990, and corresponds to the Statute of Children and Adolescents (ECA). All mothers mentioned that the test is important for the health of the child, which corroborate the literature.^6^ ^,^ ^14^ ^,^ ^16^ This examination detects diseases that could lead to neurological delay, among other changes. Thus, upon diagnosis, it is possible to establish the early treatment, thereby preventing damage to the newborn.

An interesting finding of the study was the fact that approximately 63% of the puerperal women would like to receive further information about the Guthrie test, with emphasis on the period appropriate for the sample collection, followed by the diseases screened. This finding is consistent with a previous study, which showed that 44% of the participants would like to have more information about the subject.^6^ In our study, despite not knowing the appropriate period, the majority of participants (85.9%) took their children for the test between the third and seventh day of birth, as recommended by the Ministry of Health. This situation also occurred with mothers who went to the Municipal Health Centers of Rio de Janeiro.^15^ One possible explanation for this result is the guidance provided by health professionals when they were discharged from the hospital after delivery. This guidance consisted of alerting the mothers to take their children for the test as soon as possible, without however specifying the appropriate period.^18^ The mother’s unawareness about the appropriate time for the completion of the test is evident in the scientific literature.^16^ ^,^ ^17^ ^,^ ^18^ ^,^ ^19^ ^,^ ^20^ However, in relation to the information on the Guthrie test, a recent study conducted in Rio Grande do Sul showed that mothers would like to know why the Guthrie test is carried out, how the test procedure would be and what are the diseases detected.^18^

In our study, mothers did not know what are the diseases investigated by the Guthrie test. These data corroborate those already seen in the literature.^6^ ^,^ ^16^ ^,^ ^18^ ^,^ ^20^ A study conducted at the Municipal Health Center of Rio de Janeiro showed that 40 (80%) women who were interviewed did not know the names of the diseases screened, whereas 5 of them (10%) knew the name of at least one disease and the other 5 (10%) reported wrong diseases.^20^ The diseases that the women knew the most about were the Hb and the PKU. A recent study conducted in São Carlos, a city in the interior of São Paulo state, Brazil, with 119 mothers of children with altered screening test for Hb, showed that 96.6% (n=15) of them were informed about the adequate period for the sample collection for the test and only 4 (3.4%) were informed about the diseases that would be investigated and about the risks of not performing the test.^21^ This same study analyzed the information received by mothers in primary health care, at the time of sample collection for the Guthrie test. Of the total of 119 mothers, only 34 were informed of how the test was performed and 12 of them, on the characteristic of the screening, which implies the need to carry out further tests.^21^

Regarding to the purpose of the test, in this study, a significant number of interviewees replied that the test aimed at detecting a disease. This suggests that mothers do not know that the majority of the diseases investigated bear genetic etiology and that, therefore, if any disease is detected, there is a risk of recurrence among siblings. This fact was also observed in a study conducted in the city of Cáceres, Mato Grosso, Brazil, in which the participants have associated the Guthrie test with the discovery of diseases in general.^17^

The mothers investigated, emphasized the importance of the Guthrie test to detect diseases for which there is treatment. This finding was very important, considering that the NNSP was established to identify early incurable diseases, but they have a good prognosis if diagnosed and treated in the neonatal period. The main goal of the NNSP is the prevention and reduction of morbidity and mortality caused by diseases screened.^2^ ^,^ ^4^ A survey with 55 multiparous mothers in the state of Mato Grosso, showed that a small group (6.1%) associated the test with the discovery of diseases and the completion of treatment, but with a focus on the cure.^17^ In our study, only one mother stated that the test prevents neurological delay, that is, the preventive characteristic of the Guthrie test was therefore compromised.

The majority (91%) of the puerperal women responded that the Guthrie test is performed by means of collecting blood from the foot of the newborn. This result was expected due to the name of the examination. A few mentioned the possibility of blood collection by venipuncture. In this sense, it is necessary to clarify the possible locations and forms for sample collection of biological material.

In this study, the healthcare professional who communicated more about the Guthrie test was the doctor, a finding that is consistent with the literature.^15^ ^,^ ^19^ ^,^ ^22^ Delvivo et al.,^22^ for example, showed that the guidelines were given by medical professionals in 77% of cases, and by nurses, in 23% of them. An alarming result was the fact that only 3% of those mothers received adequate guidance on the Guthrie test. However, a recent survey showed that, in 93.3% of cases, the nurse was the health care professional responsible for the provision of information about neonatal screening to mothers, and the physicians were responsible for only 5.9% of the cases.^21^ In another study in the city of Cáceres, Mato Grosso, Brazil, 51% of mothers reported having received information from nurses and 22.4% of them from physicians.^17^

As for the orientation on the Guthrie test, the ideal is to be performed during the prenatal, because during this period the pregnant woman has more available time, is more attentive, and is able to better assimilate information. In the puerperal period, the woman is more concerned with the care of the neonate, such as feeding, hygiene, vaccination, and the adaptation to accommodate a new family member. At that time, the puerperal women live different emotions that may lead to an overload, and which may influence the guidance provided after discharge from the hospital. In our study, the guidance occurred primarily in the prenatal period (57%), and secondly at the time of discharge (43%). A qualitative study with seven primiparous puerperae showed that the little and brief information they received were provided in the maternity ward.^18^ Regarding the prenatal period, it is advisable to check the approach of this theme in the consultations due to its extreme importance for neonatal health. The puerpera investigated, considered good quality of information received. Despite this result, the knowledge that was presented was superficial. A study carried out in Juiz de Fora, Minas Gerais, showed that only 3% of mothers received guidelines which were considered complete, and 62% rated the information as incomplete and/or misleading.^22^

It is also worth noting that recent studies on neonatal hearing screening were performed with nursing professionals^23^ and mothers,^24^ addressing an approach similar to this study. A quantitative study with 80 mothers of infants who underwent the ear test for their children showed that almost all of them considered the test important, and were instructed to perform it; however, 51% did not know what the test would detect. The health professionals responsible for the guidance were the physicians (53%) and nurses (44%), which corroborates the data of our study.^24^

In this study, women with higher schooling had a greater knowledge of the purpose and importance of the Guthrie test. Although there is a relationship between education and income, this has not had significant influence in the analysis. Multiparous women could demonstrate greater knowledge on the subject; however, in our study, the majority of them were primiparous (approximately 70%). However, the data from this study emphasizes the need for greater guidance to mothers about the Guthrie test, as it represents the greatest preventive pediatrics action related to genetics around the world. In Brazil, mothers who have good knowledge on the subject, contribute to the effectiveness of neonatal screening. This research is of significant relevance to the child’s health, because it is related to research on diseases among newborns.

The limitations of this study were the sample by convenience and the non-probabilistic sample. However, we presented a description of how the sample was obtained aiming at addressing the credibility of the findings. Added to this, a statistical power above 80% ensures the reliability of the findings presented. The questionnaire was not submitted to the pretest or pilot study, but appreciated by our research group in order to verify its applicability.

In summary, mother’s knowledge about the Guthrie test is superficial and may be a consequence of the poor performance of the health care team. Our data emphasize the need to prioritize permanent education actions in health services that focus on the neonatal screening, thereby aiming to improve the quality of the care provided to the mother and the newborn.
